# Urbanization, Human Inequality, and Material Consumption

**DOI:** 10.3390/ijerph20054582

**Published:** 2023-03-04

**Authors:** Shuai Zhang, Dajian Zhu, Lilian Li

**Affiliations:** 1 College of Design and Innovation, Tongji University, Shanghai 200092, China; 2 School of Economics and Management, Tongji University, Shanghai 200092, China; 3 School of Economics, Jiangxi University of Finance and Economics, Nanchang 330013, China

**Keywords:** urbanization, human inequality, material consumption, coefficient of human inequality, material footprint

## Abstract

Global material consumption needs to be reduced to be within its planetary boundary. Urbanization and human inequality are two profound economic-social phenomena, which have potential impacts on material consumption. This paper aims to empirically explore how urbanization and human inequality affect material consumption. For this aim, four hypotheses are proposed and the coefficient of human inequality and material footprint per capita are employed to measure comprehensive human inequality and consumption-based material consumption, respectively. Based on an unbalanced panel data set of around 170 countries from 2010 to 2017, the regression estimations demonstrate that: (1) urbanization reduces material consumption; (2) human inequality increases material consumption; (3) the interaction effect between urbanization and human inequality reduces material consumption; (4) urbanization reduces human inequality, which explains why the interaction effect works; (5) urbanization makes more sense for reducing material consumption if the extents of human inequality are larger and the positive impacts of human inequality on material consumption are weakened if the extents of urbanization are larger. It is concluded that promoting urbanization and reducing human inequality are compatible with both ecological sustainability and social fairness. This paper contributes to understanding and achieving the absolute decoupling between economic-social development and material consumption.

## 1. Introduction

Ecological impacts are beyond ecological limits in general and, hence, need to be reduced to be sustainable [1]. In addition to carbon emissions, material consumption is another important aspect of ecological impacts [2,3]. Carbon emissions are the most typical high-entropy waste, whereas material consumption is the most typical low-entropy material and resource (including energy). Ecological consumption is related to consumption but is not the same as consumption. To be simple, material consumption is the low-entropy material and resource used behind consumption. Along with economic-social development and technological progress, material consumption is likely to be decoupled from consumption in a relative or absolute way. Relative to the extensive research on carbon emissions, research on material consumption is less, especially empirical research on exploring influencing economic-social factors of material consumption. Since 1970, global material consumption has quadrupled [4]. Global material consumption needs to be reduced to be within its planetary boundary. It should be stressed that material consumption is on a per capita basis throughout the whole paper. Even though the issue of population is important and critical for reducing and keeping global material consumption within its planetary boundary, this paper considers that the research on material consumption per capita makes more sense for exploring pragmatic and realistic ways of reducing material consumption and achieving ecological sustainability [5].

Urbanization and human inequality are two important and widespread socioeconomic phenomena which have profound impacts on many aspects of residents’ daily lives. Urbanization is an inevitable, ongoing, and accelerating trend around the whole world, which brings about a lot of changes and new life chances to a great many of residents [6,7]. Human inequality is a serious and prevailing socioeconomic problem, which exerts profound negative impacts on residents’ well-being, such as undermining economic productivity, increasing violence, worsening social cohesion, and decreasing social mobility [8,9,10,11]. The reasons for using the term human inequality are that it denotes comprehensive inequality and that it is related to the concept of human development proposed by the United Nations Development Programme (UNDP) in the well-known and consecutive Human Development Reports. Human development aims to expand the richness of human life and includes dimensions such as income, health, and education. The reasons are further elaborated below. Because urbanization and human inequality are just around us and integrated into every aspect of our daily lives, they are both thought to be potential influencing factors for material consumption [10]. Theoretically and empirically, this paper aims to explore how urbanization and human inequality affect material consumption, which enriches the research on material consumption and its influencing factors.

For the past several decades, billions of rural residents globally have left their original homeland and moved to urban areas for work, education, healthcare, their children, etc. In the near future, more and more rural residents will immigrate into and dwell in urban areas, especially in developing and populous countries such as China, India, and Ethiopia [12,13,14,15]. It is expected that at least two-thirds of the global population will be city dwellers by 2050 [16]. Two-thirds by 2050 is a rather conservative estimate of the global urbanization process. Because of different physical infrastructures, social and cultural norms, and population densities, living in urban and rural areas implies different lifestyles and consumption habits, which might generate obvious impacts on material consumption [17]. In addition, urbanization is likely to affect other economic-social phenomena (for example, human inequality), which might have significant impacts on material consumption [18]. In this way, urbanization might also indirectly affect material consumption. Therefore, it is meaningful to explore how urbanization affects material consumption. Would the ongoing urbanization help reduce material consumption? Or is the trend of urbanization compatible with ecological sustainability?

Human inequality is not we want, but it exists everywhere. Human inequality cannot be eliminated but it can be reduced. Among the SDGs of the United Nations, SDG 10 (Reduced Inequalities) urges countries to reduce human inequality in a comprehensive and timely manner [19]. Income inequality has been referred to too often among the literature which explores the relationships between human inequality and ecological impacts, such as Hausman and Stolper [20], Kocak and Baglitas [21], Kopp and Nabernegg [10], and Andersson [22]. In fact, human inequality includes not only income inequality but also some other important inequalities in human development, such as inequality in education and health care [8,23].

Although inequalities in human development and income inequality are closely related, they mean different scales and connotations for human inequality. For more than one third of countries, inequality in education, health care, or both is larger than inequality in income; for countries with lower levels of human development, human inequality tends to be greater in more dimensions [8,24]. Despite the fact that humans as a whole have made tremendous progress in advancing human development, for example, by reducing extreme poverty and deprivations, inequalities in human development still remain widespread and have been notable roadblocks to fully achieving the SDGs and systematically improving residents’ quality of life [8]. Human development and inequalities in human development are negatively and strongly correlated, which suggests that reducing inequalities in human development would improve human development in a significant manner [8,24]. Therefore, in this paper, human inequality refers to inequalities in human development rather than income inequality. Income inequality is just one dimension or one part of human inequality.

More evidence demonstrate that human inequality contributes to enlarging ecological impacts and worsening environmental conditions [19]. By virtue of social comparisons and the demonstration-imitation effect, human inequality is likely to make a difference in residents’ choices of consumption habits and lifestyles, which might influence material consumption. Human inequality might also cause and strengthen unequal distributions of opportunities, rights, and power, which tend to be inconsistent with ecologically-oriented public policies and legislation [8]. From the perspective of ecological sustainability, what is the cost of human inequality? Would human inequality bring about more material consumption? Or would efforts for reducing human inequality also help reduce material consumption?

What is more, as two of the most important economic-social phenomena in the process of human development, urbanization and human inequality cannot be independent from each other. Urbanization affects both rural and urban residents. For residents involved in urbanization actively or passively, their trajectories of human development are likely to be changed and the opportunities and chances of a better life are likely to be different and increased, which might have significant impacts on human inequality at the national level. As a result, urbanization might have significant impacts on human inequality and the potential impacts of urbanization and human inequality on material consumption might not be isolated. Therefore, the interaction effect between urbanization and human inequality on material consumption needs to be explored. Would the interaction effect reduce material consumption? If the interaction effect makes a difference for material consumption, what is the working mechanism? Would the impacts of urbanization and human inequality on material consumption be different if the other factor is considered?

In order to estimate how urbanization and human inequality affect material consumption, this paper puts forward four hypotheses, which are to be presented in Section 2. In a detailed way, the four hypotheses describe the potential impacts of urbanization and human inequality on material consumption. Based on an unbalanced panel data set and regression estimations, this paper empirically tests the four hypotheses. The empirical results are useful for understanding the potential impacts and designing evidence-based and scientific ways of reducing material consumption.

For the regression estimations of the four hypotheses to be valid, the selection of appropriate indicators of urbanization, human inequality, and material consumption is the most important precondition. Consistent with previous research such as Amer et al. [25] and Kwakwa et al. [26], urbanization is measured by the urban population (% of total population) (URB). Larger portions of residents living in urban areas indicate larger extents of urbanization. For countries with increasing populations, larger extents of urbanization imply increasing urban populations; for countries with decreasing populations, larger extents of urbanization do not necessarily imply increasing urban populations. On the global level, because global population has been increasing over the past decades, larger extents of urbanization imply increasing urban populations. The urbanization process is the redistribution of the population between rural areas and urban areas. Living in urban areas is accompanied by urban lifestyles and consumption habits.

This paper employs the coefficient of human inequality (CHI), which was proposed by the UNDP in 2010, to measure human inequality [24]. CHI is the percentage loss in the Human Development Index (HDI) due to inequality in income, inequality in education, and inequality in health. CHI is the arithmetic mean of inequality in income (%), inequality in education (%), and inequality in life expectancy (%). The composition of CHI further demonstrates that income inequality is only one part or one dimension of human inequality and that human inequality includes at least economic inequality (inequality in income) and social inequality (inequality in education and life expectancy). Larger CHI values indicate larger extents of human inequality. By employing CHI, human inequality indicates inequality of capabilities [8]. Based on HDI and CHI, the inequality-adjusted Human Development Index is constructed [24,27].

Material footprint per capita (MF) is employed to measure material consumption. MF was put forward by Wiedmann et al. [28] and measures raw material and energy (including biomass, construction minerals, fossil fuels, metal ores, etc.) extracted to fulfill the final demand of an economy [29,30,31]. As a typical consumption-based, supply-chain-wide, and trade-adjusted ecological indicator, MF presents a relatively accurate picture of material consumption in the globalized economies and is closely correlated with residents’ consumption preferences and living habits [3,32]. MF is robust against outsourcing, which means that the separation of production and consumption does not influence the measurement of real material consumption and that a country could not simply reduce material consumption by relocating material-intensive production to other countries, such as China and Vietnam [4]. MF is employed as a core indicator for monitoring progress towards achieving SDG 8 and 12 and is considered to be used in the monitoring framework of the circular economy [4,33,34]. Based on MF and carbon emissions, Hickel constructed the Sustainable Development Index [35], the UNDP constructed the Planetary Pressures-adjusted HDI [3], and Zhang and Zhu constructed the Planetary Boundaries-adjusted HDI [36].

For 2016 to 2030, sustainable development is the ultimate goal of public policies of the United Nations. The basic and fundamental requirement of sustainable development is to decouple economic-social development from ecological impacts in an absolute way, i.e., improving economic-social performance with a reduction in ecological impacts [4,37]. The absolute decoupling is also required for humanity to finally live in a socially just and ecologically safe space [38,39]. Promoting urbanization and reducing human inequality are two important aspects of economic-social development. By combining urbanization, human inequality, and material consumption into one identical framework, our research aims to make a contribution to understanding and achieving absolute decoupling.

To be specific, the contribution of this paper is threefold: first, research rarely employs CHI to explore the relationships between human inequality and ecological impacts and our research fills this gap; second, this paper explores the interaction effect between urbanization and human inequality on material consumption and explains its working mechanism; third, this paper explores whether both advocating urbanization and reducing human inequality generate two dividends, i.e., encouraging ecological sustainability and promoting social fairness.

The remainder of the paper is organized as follows. Section 2 provides the four hypotheses to be tested. Section 3 presents the regression variables, data sources, and data description. Section 4 shows the estimation results of the four hypotheses. The Discussion and Conclusions are presented in Section 5 and Section 6, respectively.

## 2. Four Hypotheses to Be Tested

According to the research questions in the Introduction, four respective hypotheses are put forward. Hypothesis three and four are dependent on hypothesis one and two. Hypothesis four tries to explain why hypothesis three works. The four hypotheses and their corresponding reasons are as follows.

### 2.1. Hypothesis One: Urbanization Is Expected to Reduce Material Consumption

As the ecological modernization theory suggests, because of economies of scale, the agglomeration effect, better stewardship, and more advanced technologies, transportation, infrastructure, sanitation, electricity, heating, cooling, etc. are more ecologically efficient in urban areas [30,40,41]. For example, in urban areas, residents are more likely to live in apartments and take public transit to work, which are much more ecologically efficient than living in houses and taking private cars to work, respectively. Ideally, compact and integrated cities have the potential to shorten commuting distances and reduce transport needs, which reduces urban material consumption [42,43]. The sizes of dwellings in urban areas are usually smaller, which is correlated with less material consumption [33]. What is more, new and ecologically friendly forms of economies, such as the circular economy and the sharing economy, are more likely to be put into practice on a large scale in urban areas. In brief, high-density and compact lifestyles and consumption habits and modern vertical buildings are expected to make material consumption less intensive and more efficient in urban areas [44,45]. On the other hand, urbanization is also strongly correlated with more residents with higher levels of human development, which is the driving force of increasing material consumption. This is the other side of the urbanization coin. However, this does not indicate that the negative impacts of the above urban characteristics on material consumption could be counteracted. Thanks to the above urban characteristics, urbanization has the potential to decouple economic-social development (human development) from ecological impacts in an absolute way. Therefore, urbanization is expected to reduce material consumption. 

### 2.2. Hypothesis Two: Human Inequality Is Expected to Increase Material Consumption

Human inequality is correlated with residents’ consumption and living preferences [46]. In societies with larger extents of human inequality, residents are more likely to ignore and more reluctant to adopt ecologically efficient consumption choices and living habits. The residents of areas with higher levels of human development usually enjoy higher levels of material consumption, such as more resource-demanding food and drinks and long-distance air travels; higher levels of human development do not automatically generate more consciousness and responsibilities for reducing material consumption [19,47,48]. Driven by increasing vanity, conspicuous consumption, and the continuous pursuit of relative social status, the residents of areas with lower levels of human development usually try their best to imitate and follow material-intensive consumption choices and living habits enjoyed by residents of areas with high levels of human development [10,49]. To a certain extent, the lifestyles and consumption patterns are mainly dominated by the residents of areas with higher levels of human development, who are often taken as ‘role models’ [17]. If the residents of areas with higher levels of human development do not take the lead in reducing material consumption in their daily lives, it is unfair and unrealistic to demand and urge the residents of areas with lower levels of human development to give up following and pursuing material-intensive lifestyles and consumption choices.

All in all, residents who live in societies with larger extents of human inequality, tend to possess more, buy more, and consume more (over-consumption) rather than act according to the principles of sufficiency and ecological sustainability. On the contrary, because residents, who live in more equal societies are less bothered and influenced by relative status and vanity, they are more likely to be ecologically conscious and to adopt materially less-intensive lifestyles and consumption habits.

Human inequality is also related with some public policies which are conducive to the material-intensive consumption of goods and services and more unlikely to pursue long-term goals of ecological sustainability. Human inequality depresses political participation by the residents of areas with low levels of human development. In societies with larger extents of human inequality, power and policy-making processes are mainly dominated and manipulated by some privileged classes in their favor [8,24]. Because they benefit more and suffer less, the privileged classes usually prefer material-intensive goods and services. What is more, human inequality is closely related with bad governance and corruption, which leads to less efficient management and less strict regulation of material consumption [8,10]). On the contrary, in societies with smaller extents of human inequality, power is more equally distributed and policy-making processes are more transparent and democratic, so ecologically oriented voices and opinions are more easily heard and adopted and material-intensive consumption activities can be avoided to a certain extent. In conclusion, ecologically oriented and long-term public policies are more compatible with more equal societies.

To sum up, human inequality is expected to be positively correlated with material consumption. In this sense, hypothesis two is called the ‘Material trap of human inequality’ in this paper. Accordingly, human inequality is not desired not only from the perspective of social fairness but also from the perspective of ecological sustainability.

### 2.3. Hypothesis Three: The Interaction Effect between Urbanization and Human Inequality Is Expected to Reduce Material Consumption

Economic factors, for example, the transition of economic structure, mainly determine the speed and scale of urbanization. Within a country, the expansion of industry and service sectors, which usually concentrate in urban areas, push and attract more and more rural residents to move to urban areas. Because the lives of a great many residents have been and will be changed by urbanization, the urbanization process might make a difference towards human inequality at the national level [11,18]), which is elaborated on in hypothesis four. Although social factors, for example, human inequality, might also affect residents’ decisions for living in rural or urban areas, it is assumed that their impacts are not decisive. For most rural residents, the reasons for choosing to move to urban areas and being able to have a decent life in urban areas are mainly related to more job opportunities and higher levels of income rather than human inequality. The expectation of a better life induces rural residents to leave for urban areas, which in turn might reduce national human inequality. In this sense, it is inferred that following the effect of urbanization, the interaction effect between urbanization and human inequality is expected to reduce material consumption.

### 2.4. Hypothesis Four: Urbanization Has Indirect Negative Impacts on Material Consumption by Reducing Human Inequality

Generally speaking, there is an obvious gap in human development between urban and rural areas [18]. Urban areas are the gathering places of creative corporations, high-quality schools and universities, leading general and specialized hospitals, etc., which are foundations and necessary conditions for advanced human development. Urbanization promotes rural-urban migration, and more and more rural residents become urban residents who usually enjoy higher levels of human development [50,51]. When the rural residents settle in urban areas, they are able to have more chances at having jobs of higher salaries, at getting high-quality educations for themselves and their children, at receiving better healthcare at all levels, etc. [16,18].

What is more, because of less competition and overcrowding, the remaining residents of less rural areas are likely to have more material and non-material resources and opportunities for their human development [51,52]. Therefore, in a gradual and logical fashion, urbanization increases the number of residents of areas with higher levels of human development in urban areas and decreases the amounts of the residents of areas with lower levels of human development in rural areas. Urbanization is likely to help narrow the urban-rural gap of human development at the national level. Accordingly, urbanization is expected to help reduce human inequality, by which urbanization indirectly reduces material consumption. By this working mechanism, hypothesis three works.

## 3. Regression Variables, Data Sources and Data Description

As we discussed above, in order to conduct regression estimations of the four hypotheses, URB is used to measure urbanization, CHI is used to measure human inequality, URB × CHI is used to measure the interaction effect between urbanization and human inequality and MF is used to measure material consumption. According to the above four hypotheses, MF, URB, CHI and URB×CHI are core regression variables.

According to previous research, some other variables are employed as control variables of the regression estimations. Because the Environmental Kuznets Curve (EKC) depicts an inverted U-shaped relationship between economic growth and ecological impacts, this paper employs Gross Domestic Product per capita (GDPPC) to measure economic growth and quadratic of GDPPC is also employed as a control variable [45,53]. As two important dimensions of human development, education and health are likely to affect material consumption. The residents of areas with different levels of education and health might have different living styles and consumption habits. The mean years of schooling (MYS) is employed to measure education levels and the life expectancy at birth (LEB) is employed to measure medical and health levels. Because inflation has some impacts on consumption behaviors and expectations, which might affect material consumption, consumer prices (annual %) (CPs) are employed as control variables. Economic structures are correlated with residents’ work choices, income levels, and consumption habits, which are likely to affect material consumption. Services, value-added (% of GDP) (SER) and industry, value-added (% of GDP) (IND) are employed to measure economic structures.

Even though material consumption is on a per capita basis throughout the paper, population factors are likely to affect the relationships between urbanization, human inequality, and material consumption. Population density (PD) is selected as a typical proxy of population and is expressed as midyear population/land area. Because national land areas are fixed in general, PD has the same trends with national population sizes. In addition, the balance of males and females is also a typical population factor. Therefore, gender balance (GB) is employed as a control variable and is expressed as the absolute value of females (% of total population) minus 50%. For the globalized economies, because residents have more goods and services to choose from to buy and more job opportunities, trade has the potential to affect material consumption. Trade openness (TO) is employed as a typical indicator of trade and is expressed as imports of goods and services (% of GDP) plus exports of goods and services (% of GDP). 

Data for MF were obtained from the United Nations Environment Programme. (The website is https://environmentlive.unep.org/downloader, accessed on 8 March 2022). The data source provides the data for national MF from 1990 to 2017. Data for CHI and MYS were obtained from the Human Development Data Center of the UNDP. (The website is http://hdr.undp.org/en/data, accessed on 22 December 2022). This data source provides the data for national CHI from 2010 to 2021 and the data for national MYS from 1990 to 2021.

The data for GDPPC, LEB, URB, CP, SER, IND, TO, PD, and GB were obtained from the World Bank Open Data. (The website is https://data.worldbank.org/indicator, accessed on 22 December 2022). This data source provides the data for national GDPPC from 1990 to 2021, the data for national LEB from 1960 to 2020; the data for national URB, CP, SER, IND, TO, and GB from 1960 to 2021; and the data for national PD from 1961 to 2020.

After sorting out all of the above data for the variables, an unbalanced panel data set of around 170 countries from 2010 to 2017 was employed for the regression estimations. Because CHI was just proposed in 2010, the CHI data limits the research period. Data statistics and the descriptions of the regression variables are show in Table 1.

According to O’Neill et al. [54], the planetary boundary of MF is 7.20 t and the MFs of only 44% of countries are within the planetary boundary. For the data sample in this paper, MFs of less than half (42.94%) of all the observations were within the planetary boundary (7.20 t), which is consistent with O’Neill et al. [54]. For example, in the latest year, 2017, the MFs of only 72 countries (42.35% of the whole sample of countries) were within the planetary boundary.

There are significant national differences in urbanization rates and human inequality. For the average URB during the research period, the URB values for Burundi, Papua New Guinea, Malawi, Niger, and Rwanda were below 17% and the URB values for Singapore, Kuwait, Qatar, Belgium, and Uruguay were over 94%. For the average CHI during the research period, the CHI values of Czechia, Slovenia, Iceland, Finland, and Norway were the smallest, all of which were no more than 6.22%; the CHI values of the Central African Republic, Sudan, Haiti, Sierra Leone, and Nigeria were the largest, all of which were no less than 38.39%; for the three most populous countries, the CHI of India was 27.54%, which was much larger than that of China (21.20%) and the United States (13.46%).

## 4. Estimation Processes and Results

This section first tests hypotheses one through three. Then, hypothesis four is tested. This section further conducts a sensitivity analysis of the regression estimations. Based on the GDPPC levels, this section finally conducts regression estimations for two sub-sample countries.

### 4.1. Benchmark Estimations: Testing Hypothesis One–Three

In order to empirically test hypotheses one through three, the following econometric model is proposed.

(1) $$MF_{it}=\beta URB_{it}+\lambda CHI_{it}+\gamma CHI_{it}\times URB_{it}+\upsilon_{k}CV_{it,k}+\delta_{i}+{ο}_{t}+\varepsilon_{it}$$

In Model (1), *CV_it,k_* is the set of control variables; *k* is the number of control variables; *i* and *t* are the country and year, respectively; $\delta_{i}$ and ${ο}_{t}$ are the country fixed effects and year fixed effects, respectively; and $\varepsilon_{it}$ is the random error term. 

To obtain the net impacts of urbanization and human inequality on material consumption, their marginal effects are calculated, respectively.

(2) $$\partial MF_{it}/\partial URB_{it}=\beta +\gamma CHI_{it}$$

(3) $$\partial MF_{it}/\partial CHI_{it}=\lambda +\gamma URB_{it}$$

β reflects the impact of urbanization on material consumption with an expected negative sign; λ reflects the impact of human inequality on material consumption with an expected positive sign; γ reflects the impact of the interaction effect between urbanization and human inequality on material consumption. If γ<0, it indicates that urbanization weakens the assumed positive impact of human inequality on material consumption, i.e., the interaction effect has a dampening effect on human inequality and human inequality reinforces the assumed disincentive effect of urbanization on material consumption, i.e., the interaction effect has a promotional effect on urbanization. On the contrary, if γ>0 it indicates that urbanization strengthens the assumed positive impacts of human inequality on material consumption and that human inequality weakens the assumed negative impacts of urbanization on material consumption. Prior to the regression estimations, we calculate variance inflation factors (VIFs) to test whether the econometric problem of multi-collinearity of the main explanatory variables exists. Table 2 shows the VIF values. The VIF values of the explanatory variables are distinctly less than 10, which indicates that there is no significant multi-collinearity among the explanatory variables.

In order to judge which regression method (the fixed effects model or the random effects model) is to be employed, the Hausman test is conducted. The results of the Hausman test are shown in Table 3. Column (1) shows the estimated coefficients of the explanatory variables of the fixed effects model (FE). Column (2) displays the estimated coefficients of the explanatory variables of the random effects model (RE). The Chi-square statistic of the Hausman test is 119.16 and is significant at the 1% level, which indicates that the fixed effects model is more appropriate to be employed in the benchmark regressions.

We adopt the fixed effects model to estimate Model (1) and conduct a series of robust identification strategies to verify the regressions. The main estimation results are provided in Table 4. Column (1) examines the impacts of urbanization on material consumption separately by using country and year fixed effects, which eliminates potential omitted variables from the country and year levels. Column (2) adds human inequality to Column (1) and estimates the impacts of urbanization and human inequality on material consumption. Based on Column (2), Column (3) incorporates the interaction effect between urbanization and human inequality. Column (4) further adds GDPPC and the quadratic of GDPPC into the regressions to estimate whether economic levels and material consumption follow the law of the EKC. Column (5) further considers the other control variables at the national level, which are used as the final analysis.

First, the effect of urbanization on material consumption is significantly negative at the 1% level, with an estimated coefficient of −0.087, which indicates that a unit increase in the level of urbanization results in a 0.087 unit reduction in material consumption. Hypothesis one is verified.

Second, the effect of human inequality on material consumption is significantly positive at the 5% level, with an estimated coefficient of 0.073, which indicates that a unit increase in human inequality increases material consumption by 0.073 units. Hypothesis two is validated.

Third, the interaction effect between human inequality and urbanization is significantly negative at the 5% level, with an estimated coefficient of −0.001. Hypothesis three is confirmed.

According to Equations (2) and (3), the marginal impacts of urbanization on material consumption are −0.087–0.001 CHI, which means that the negative impacts of urbanization are more profound if the extents of human inequality are larger; the marginal impacts of human inequality on material consumption are 0.073–0.001 URB, which means that the positive impacts of human inequality on material consumption are weakened if the extents of urbanization are larger.

In addition, we find that: (1) the EKC hypothesis is verified and the relationship between economic growth and material consumption is also an inverted U-shape; (2) consumer prices, industry, value-added and population density are negatively related with material consumption; and (3) trade openness, life expectancy at birth and gender balance are positively related with material consumption. All of the above findings are consistent with most of the previous research.

### 4.2. One Mechanism Estimation: Testing Hypothesis Four

Hypothesis four assumes that urbanization reduces human inequality, which explains why the interaction effect between urbanization and human inequality has a negative impact on material consumption. To test the working mechanism, Models (4) and (5) are established. If we substitute Model (4) into Model (5), we obtain Model (6).

(4) $$CHI_{it}=\beta URB_{it}+\upsilon_{k}CV_{it,k}+\delta_{i}+{ο}_{t}+\varepsilon_{it}$$

(5) $$MF_{it}=\beta^{\prime }URB_{it}+\tau CHI_{it}+\upsilon_{k}^{\prime }CV_{it,k}+\delta_{i}+{ο}_{t}+\varepsilon_{it}$$

(6) $$MF_{it}=(\beta^{\prime }+\tau \beta )URB_{it}+(\upsilon_{k}^{\prime }+\upsilon_{k}\tau )CV_{it,k}+\delta_{i}+{ο}_{t}+\varepsilon_{it}$$

Model (6) is the total effect estimation of the impacts of urbanization on material consumption. If all of β, $\beta^{\prime }$, τ, and $\beta^{\prime }+\tau \beta$ pass the significance tests, this indicates that urbanization also affects material consumption through the mechanism channel of human inequality. It is concluded that $\beta^{\prime }$ denotes the direct impacts of urbanization on material consumption and that τβ denotes the indirect impacts of urbanization on material consumption (the mechanism effect).

The estimation results of Model (4–6) are displayed in Table 5. Column (1) demonstrates that urbanization significantly reduces human inequality. Column (2) verifies that urbanization significantly reduces material consumption, and that human inequality significantly increases material consumption. Column (3) demonstrates that the total effect of urbanization on material consumption is significantly negative. Moreover, Columns (4–6) add the control variables to Columns (1–3), respectively, and the estimated impacts are still statistically significant. The estimation results in Columns (4–6) verify that urbanization reduces material consumption indirectly through the channel of human inequality with a coefficient of −0.004 and a direct effect with a coefficient of −0.153.

### 4.3. Further Sensitivity Analysis

In order to verify the robustness of the benchmark estimations, we adopt the following strategies. The details are shown in Table 6. Column (1) takes a 1% reduced-tail treatment of the dependent variable. Column (2) re-estimates the benchmark model by replacing GDPPC with GNIPC (gross national income per capita). Data for GNIPC were obtained from the World Bank Open Data (https://data.worldbank.org/indicator, accessed on 22 December 2022). Based on Column (2), Column (3) takes a 1% reduced-tail treatment for the dependent variable. Column (4) takes a 1% reduced-tail treatment for the core regression variables (the dependent variable, URB, CHI, and URB × CHI). The results in Table 6 prove that the benchmark estimations are valid and robust.

### 4.4. Sub-Sample Estimations

The impacts of urbanization and human inequality on material consumption might be different for different sub-sample countries. Based on the GDPPC levels, this paper divides the whole sample countries into the low-GDPPC countries sample and the high-GDPPC countries sample. For classification, the threshold value of GDPPC is USD 11.86912 thousand. Table 7 presents the estimation results of the impacts of urbanization and human inequality on material consumption for the two sub-sample countries. Columns (1–2) show the estimation results of the low-GDPPC countries sample and the high-GDPPC countries sample, respectively. Following the methods of the sensitivity analysis in Section 4.3, Columns (3–4) take a 1% reduced-tail treatment for the dependent variable of the low-GDPPC countries sample and the high-GDPPC countries sample, respectively, and Columns (5–6) replace the GDPPC with GNIPC of the low-GDPPC countries sample and the high-GDPPC countries sample, respectively. Columns (3–4) and Columns (5–6) demonstrate that the estimation results of the two sub-sample countries are valid.

For the low-GDPPC countries sample, the estimated results are consistent with those of the whole sample countries and the estimated coefficients of URB, CHI, and URB × CHI do not have large differences from those of the whole sample countries. In contrast, for the high-GDPPC countries, although urbanization is still statistically significant, human inequality and the interaction effect are not significant influencing factors of material consumption. For the high-GDPPC countries sample, the negative impacts of urbanization on material consumption are much more profound than those of the low-GDPPC countries sample and the whole sample countries.

## 5. Discussion

The regression estimations show that urbanization is a significant factor for reducing material consumption, which is consistent with most of the previous research, such as Charfeddine and Mrabet [55], Nathaniel et al. [56], Amer et al. [25], and Kwakwa et al. [26]. Urbanization itself is conducive to achieving ecological sustainability. What is more, it is empirically proven that urbanization helps reduce human inequality. From the perspective of ecological sustainability, urbanization creates dual benefits, i.e., reducing material consumption directly and reducing material consumption indirectly by reducing human inequality. All in all, urbanization is compatible with both ecological sustainability and social fairness. Urbanization is one of the effective means for achieving the absolute decoupling between economic-social development and material consumption.

The regression estimations demonstrate that human inequality increases material consumption and, hence, that it is not desirable from the perspective of ecological sustainability. Therefore, reducing human inequality also creates dual benefits, i.e., promoting social fairness and encouraging ecological sustainability. The conclusions are supported by Torras and Boyce [57], Zecca and Nicolli [58], and Kocak and Baglitas [21]. Torras and Boyce proved that more equitable distributions of power result in better environmental qualities [57]. Zecca and Nicolli demonstrated that a reduction in income inequality supports eco-friendly innovations [58]. Kocak and Baglitas confirmed that reducing income inequality reduces municipal solid waste per capita in OECD countries [21]. These conclusions are in contrast with López et al. [49] and Kopp and Nabernegg [10]. By performing one redistribution simulation, López et al. found that a redistribution of income increases households’ material consumption [49]. Through a panel data set of 116 countries over 55 years, Kopp and Nabernegg (2022) demonstrated that income inequality is negatively related with carbon footprint and that reducing income inequality enlarges the ecological impacts [10]. López et al. and Kopp and Nabernegg further argued that income redistribution and ecological sustainability are hence not compatible [10,49]. Instead, our estimation results provide direct evidence that reductions in human inequality and ecological sustainability are compatible and that we have the chance of simultaneously achieving social fairness and ecological sustainability in the future. Reducing human inequality is also an effective way of achieving the absolute decoupling between economic-social development and material consumption.

The interaction effect between urbanization and human inequality is a significant factor for reducing material consumption, which makes the marginal impacts of urbanization and human inequality on material consumption interdependent. Urbanization reduces human inequality, which explains why the interaction effect is statistically significant and has negative impacts on material consumption. The working mechanism of the interaction effect is supported by Ahimah-Agyakwah et al. [59] and Wan et al. [51]. Ahimah-Agyakwah et al. demonstrated that, in sub-Saharan Africa, urbanization has a significant effect on reducing poverty, which also reduces human inequality [59]. Wan et al. argued that well-managed urbanization reduces national income inequality by narrowing the urban-rural gap [51].

It is a pity that rare research has employed CHI to measure comprehensive human inequality and to explore the relationships between human inequality and ecological impacts until now. Therefore, the above discussion of our empirical findings has to refer to the existing literature, which explores the relationships between income inequality (one dimension of human inequality) and ecological impacts. However, this does not mean that we equate human inequality with income inequality. Human inequality is much more comprehensive than income inequality.

It is also found that urbanization makes more sense for reducing material consumption if the extents of human inequality are larger and that the positive impacts of human inequality on material consumption are smaller if the extents of urbanization are larger. The significant role of the interaction effect further demonstrates that urbanization counteracts the positive impacts of human inequality on material consumption. Reducing human inequality inevitably triggers the redistribution of opportunities, rights, and power and faces obstacles set by some interest groups, whereas promoting urbanization would not in the majority of cases. Therefore, the interaction effect shows that urbanization helps us jump out of the ‘Material trap of human inequality’, even though urbanization alone is far from being enough.

The estimated results of the two sub-sample countries further support the findings derived from the estimations of the whole sample countries. For the high-GDPPC countries, human inequality and the interaction effect are not significant influencing factors for material consumption; for the low-GDPPC countries, human inequality and the interaction effect are still significant influencing factors for material consumption. One of the reasons is that the extents of urbanization are usually large in high-GDPPC countries and not large in low-GDPPC countries. For the two sub-samples, the average URB values of the high-GDPPC countries sample and the low-GDPPC countries sample were 72.98% and 42.87%, respectively. The data description of URB in Section 3 also supports the reason.

For both the high-GDPPC countries sample and the low-GDPPC countries sample, urbanization is a significant factor for reducing material consumption, which further confirms the important role of urbanization in reducing material consumption. In terms of reducing material consumption, urbanization also makes more sense for countries with larger extents of urbanization, i.e., the high-GDPPC countries sample. By means of urbanization, the high-GDPPC countries, in general, have done a better job at reducing material consumption and curing the positive impacts of human inequality on material consumption.

Low urban land use efficiency, urban sprawl, declining urban densities, deficient infrastructure, and infrastructure inequalities in some developing countries help explain why the negative impacts of urbanization on the material consumption of the low-GDPPC countries are not as profound as those of the high-GDPPC countries [11,13,45,60]). In short, uncontrolled and unplanned urbanization makes the effect of urbanization on reducing material consumption less profound [40]. In addition, as suggested by the UNDP [8,24], the average CHI values of the low-GDPPC countries sample and the high-GDPPC countries sample were 27.18% and 13.45%, respectively, which indicates that the task of reducing human inequality in the low-GDPPC countries is more difficult and arduous. The data description of CHI in Section 3 also supports this argument. Therefore, for the low-GDPPC countries, the potential and capabilities of reducing material consumption by promoting urbanization and reducing human inequality need to be further explored and improved.

## 6. Conclusions

In order to explore the relationships between urbanization, human inequality, and material consumption, this paper puts forward four hypotheses and conducts respective empirical tests. One distinguished feature of our empirical research is to employ CHI and MF to measure comprehensive human inequality and consumption-based material consumption, respectively. The regression estimations of the whole sample countries (around 170 countries from 2010 to 2017) verify the proposed four hypotheses. The regression estimations of the two sub-sample countries (the low-GDPPC countries and the high-GDPPC countries) also support the four hypotheses. By proposing and testing the four hypotheses, this paper designs a new theoretical and empirical framework for analyzing the impacts of urbanization and human inequality on material consumption.

Our empirical results imply that it is possible and realistic for humanity to achieve the absolute decoupling between economic-social development and material consumption. Advocating urbanization, reducing human inequality, and reducing material consumption are compatible in general. Furthermore, both advocating urbanization and reducing human inequality are useful for achieving both ecological sustainability and social fairness, which implies that we could kill two birds with one stone in some cases.

In order to accelerate the pace of the absolute decoupling, the policy implications of our research are quite obvious. On the global level, our research is useful for achieving SDG 10 and 12. Urbanization should be more compact, dense, and ecologically oriented and put more attention to the residents of areas with low levels of human development, so urbanization can be more effective at reducing material consumption, especially for low-GDPPC countries. Human development should be more equitable and balanced, which benefits not only social equity but also ecological sustainability. For the low-GDPPC countries, more efforts should be put into practice to reduce human inequality by a larger extent.

We provide just one mechanism to explain why the interaction effect has negative impacts on material consumption. More mechanisms need to be empirically identified to explain why the interaction effect between urbanization and human inequality works. Because the interaction effect is not very profound (the estimated coefficient is relatively small), more economic-social factors need to be identified to counteract the positive impacts of human inequality on material consumption. In addition, more empirical analyses need to be conducted to explain why human inequality and the interaction effect are not significant influencing factors for material consumption for the high-GDPPC countries sample. Because we just provide a general framework, more specific causes and examples need to be explored to explain how urbanization and human inequality affect material consumption. Most importantly, more theoretical and empirical research needs to be conducted to explore the theories for understanding the absolute decoupling and the pragmatic ways for achieving absolute decoupling.

Cities of different scales might exert different impacts on material consumption and human inequality, which is not taken into consideration by this paper. For example, cities with populations of 1 million and 10 million might have different impacts on material consumption. Detailed and empirical research on how urban scales affect material consumption is useful for designing more reasonable policies from the perspective of ecological sustainability. In addition, how the interdependencies between urban, suburban, and rural areas affect the regression estimations and our conclusions are not considered by this paper, which need to be explored in a detailed way in future research.

## Figures and Tables

**Table 1:** Data statistics and description.

Abbreviation	Variable	Obs	Mean	Std Dev	Min	Max
MF	Material Footprint per Capita	1359	13.49	15.53	0.05	121.27
URB	Urbanization	1359	58.04	22.43	10.64	100.00
CHI	Coefficient of Human Inequality	1181	20.68	10.27	4.90	44.30
GDPPC	Gross Domestic Product per Capita	1335	18.77	20.24	0.56	141.64
CP	Consumer Prices (Annual %)	1295	6.23	20.41	−11.21	388.16
IND	Industry, Value-Added (% of GDP)	1311	27.31	12.02	4.56	74.81
SER	Services, Value-Added (% of GDP)	1303	53.97	11.75	19.17	81.08
TO	Trade Openness	1288	90.31	55.80	0.20	493.88
MYS	Mean Years of Schooling	1359	8.36	3.18	1.40	14.10
LEB	Life Expectancy at Birth	1359	71.23	8.20	45.10	84.10
PD	Population Density	1359	187.32	621.146	1.75	7915.73
GB	Gender Balance	1359	1.47	3.081	0.00	26.71

**Table 2:** VIFs of the explanatory variables.

	URB	CHI	GDPPC	CP	IND	SER	TO	MYS	LEB	PD	GB
VIF	2.51	4.18	3.15	1.03	1.79	3.60	1.32	1.01	5.11	1.19	1.22
1/VIF	0.40	0.24	0.32	0.97	0.56	0.28	0.76	0.99	0.20	0.84	0.82

**Table 3:** Results of the Hausman Test of the benchmark regressions.

Variable	(1)	(2)	(3)	(4)
	FE	RE	Difference	S.E.
URB	−0.087	0.079	−0.166	0.027
CHI	0.073	0.088	−0.015	0.007
URB × CHI	−0.001	−0.002	0.001	0.000
GDPPC	0.283	0.358	−0.074	0.018
GDPPC^2^	−0.002	−0.002	0.000	0.000
CP	−0.007	−0.007	0.000	0.000
IND	−0.031	−0.028	−0.003	0.004
SER	0.000	0.012	−0.011	0.003
TO	0.005	0.011	−0.006	0.001
MYS	0.001	−0.001	0.002	0.002
LEB	0.071	0.090	−0.019	0.028
PD	−0.009	0.003	−0.012	0.002
GB	0.267	−0.175	0.442	0.067
Hausman Test	Chi^2^(10) = 119.16 ***			

Notes: *** denotes significance at the 1% level.

**Table 4:** Baseline regression estimations.

Variable	(1)	(2)	(3)	(4)	(5)
URB	−0.076 **	−0.134 ***	−0.089 ***	−0.065 **	−0.087 ***
	(0.033)	(0.031)	(0.033)	(0.032)	(0.033)
CHI		0.030 **	0.132 ***	0.059 **	0.073 **
		(0.012)	(0.029)	(0.028)	(0.030)
URB × CHI			−0.002 ***	−0.001 *	−0.001 **
			(0.001)	(0.000)	(0.001)
GDPPC				0.252 ***	0.283 ***
				(0.024)	(0.026)
GDPPC^2^				−0.001 ***	−0.002 ***
				(0.000)	(0.000)
CP					−0.007 ***
					(0.002)
IND					−0.031 ***
					(0.012)
SER					0.000
					(0.012)
TO					0.005 **
					(0.002)
MYS					0.001
					(0.007)
LEB					0.071 *
					(0.038)
PD					−0.009 ***
					(0.002)
GB					0.267 ***
					(0.101)
Constant	17.910 ***	20.156 ***	17.553 ***	13.250 ***	10.067 ***
	(1.908)	(1.833)	(1.941)	(1.922)	(3.564)
Year-fixed	Yes	Yes	Yes	Yes	Yes
Country-fixed	Yes	Yes	Yes	Yes	Yes
Obs	1359	1175	1175	1167	1080
Within R^2^	0.005	0.027	0.041	0.156	0.215

Notes: *, **, and *** denote significance at the 10%, 5%, and 1% levels, respectively; robust standard errors are reported in the parentheses.

**Table 5:** A mechanism test of the interaction effect.

Variable	(1)	(2)	(3)	(4)	(5)	(6)
	CHI	MF	MF	CHI	MF	MF
URB	−0.353 ***	−0.134 ***	−0.144 ***	−0.151 *	−0.153 ***	−0.157 ***
	(0.078)	(0.031)	(0.031)	(0.087)	(0.034)	(0.034)
CHI		0.030 **			0.028 **	
		(0.012)			(0.013)	
Constant	Yes	Yes	Yes	Yes	Yes	Yes
CV	No	No	No	Yes	Yes	Yes
Year-fixed	Yes	Yes	Yes	Yes	Yes	Yes
Country-fixed	Yes	Yes	Yes	Yes	Yes	Yes
Obs	1175	1175	1175	1080	1088	1088
Within R^2^	0.020	0.027	0.021	0.081	0.102	0.097

Notes: *, **, and *** denote significance at the 10%, 5%, and 1% levels, respectively; robust standard errors are reported in the parentheses.

**Table 6:** Robustness tests of the benchmark regressions.

Variable	(1)	(2)	(3)	(4)
URB	−0.091 ***	−0.098 ***	−0.100 ***	−0.096 ***
	(0.031)	(0.033)	(0.031)	(0.032)
CHI	0.072 **	0.073 **	0.071 **	0.081 ***
	(0.028)	(0.030)	(0.028)	(0.029)
URB × CHI	−0.001 **	−0.001 *	−0.001 *	−0.001 **
	(0.001)	(0.001)	(0.001)	(0.001)
GDPPC	0.294 ***			0.154 ***
	(0.025)			(0.017)
GDPPC^2^	−0.002 ***			−0.000 ***
	(0.000)			(0.000)
GNIPC		0.360 ***	0.365 ***	
		(0.032)	(0.031)	
GNIPC^2^		−0.003 ***	−0.003 ***	
		(0.000)	(0.000)	
Constant	Yes	Yes	Yes	Yes
CV	Yes	Yes	Yes	Yes
Year-fixed	Yes	Yes	Yes	Yes
Country-fixed	Yes	Yes	Yes	Yes
Obs	1080	1080	1080	1080
Within R^2^	0.236	0.224	0.245	0.187

Notes: *, **, and *** denote significance at the 10%, 5%, and 1% levels, respectively; robust standard errors are reported in the parentheses.

**Table 7:** Sub-sample regression estimations.

Variable	Low-GDPPC Countries	High-GDPPC Countries	Low-GDPPC Countries	High-GDPPC Countries	Low-GDPPC Countries	High-GDPPC Countries
	(1)	(2)	(3)	(4)	(5)	(6)
URB	−0.064 *	−0.286 ***	−0.064 *	−0.290 ***	−0.069 **	−0.275 ***
	(0.033)	(0.079)	(0.033)	(0.073)	(0.034)	(0.086)
CHI	0.068 **	0.029	0.068 **	0.018	0.062 **	0.130
	(0.028)	(0.130)	(0.028)	(0.120)	(0.028)	(0.159)
URB × CHI	−0.001 **	−0.001	−0.001 **	−0.000	−0.001 *	−0.002
	(0.001)	(0.002)	(0.001)	(0.002)	(0.001)	(0.002)
Constant	Yes	Yes	Yes	Yes	Yes	Yes
CV	Yes	Yes	Yes	Yes	Yes	Yes
Year-fixed	Yes	Yes	Yes	Yes	Yes	Yes
Country-fixed	Yes	Yes	Yes	Yes	Yes	Yes
Obs	549	527	549	527	550	523
Within R^2^	0.242	0.220	0.241	0.253	0.210	0.221

Notes: *, **, and *** denote significance at the 10%, 5%, and 1% levels, respectively; robust standard errors are reported in the parentheses.

## Data Availability

We have provided the details of the research data in Section 3. Researchers who are interested in the data can contact us through the correspondence email for further discussion or collaboration.
